# Supplementary material for: A novel biosensor to study cAMP dynamics in cilia and flagella
Source: eLife. 2016 Mar 22;5:e14052. doi: 10.7554/eLife.14052 (PMC4811770; doi:10.7554/eLife.14052)
Supplement: Figure 4—source data 1. — For matings, heterozygous males have been crossed with wild-type females. All data are represented as mean ± S.D., n numbers are indicated. DOI: http://dx.doi.org/10.7554/eLife.14052.008 [file elife-14052-fig4-data1.docx]

**Tables**

|  | **wild-type** | ***Prm1*-mlCNBD-FRET** |
| --- | --- | --- |
| **ratio testis weight/bodyweight** | 2.8 ± 0.7 (n = 16) | 3.0 ± 0.7 (n = 13) |
| **ratio epididymis weight/bodyweight** | 0.7 ± 0.1 (n = 16) | 0.6 ± 0.1 (n = 13) |
| **sperm count (per ml)** | 2.4 x 10^7^ ± 0.9 x 10^7^ (n = 19) | 1.5 x 10^7^ ± 0.9 x 10^7^ (n = 7) |
| **litter size**  **flagellar beat frequency (Hz)** | 6.7 ± 2.7 (n = 20)  8.4 ± 2.1 (n = 8) | 5.1 ± 2.5 (n = 11)  7.3 ± 3.0 (n = 4) |

**Figure 4 - source data 1**
