# Supplementary material for: A novel biosensor to study cAMP dynamics in cilia and flagella
Source: eLife. 2016 Mar 22;5:e14052. doi: 10.7554/eLife.14052 (PMC4811770; doi:10.7554/eLife.14052)
Supplement: Figure 5—source data 1. — Binding affinities and the cerulean lifetime are shown as mean ± S.D.; n numbers are indicated. DOI: http://dx.doi.org/10.7554/eLife.14052.010 [file elife-14052-fig5-data1.docx]

|  | **K_D_** | | **cerulean fluorescence lifetime** | |  |
| --- | --- | --- | --- | --- | --- |
| purified mlCNBD-FRET | cAMP: 66 ± 15 nM (n = 5)  cGMP: 504 ± 137 nM (n = 6) | | 0 μM cAMP: 2.38 ± 0.04 ns (n = 11)  5 μM cAMP: 2.44 ± 0.03 ns (n = 5) | |  |
| mlCNBD-FRET in HEK293 cells | cAMP: 73 ± 20 nM (n = 11) | | FLS basal: 1.88 ± 0.04 ns (n = 9)  FLS NKH/IBMX: 1.98 ± 0.03 ns (n = 9)  FLIM basal: 2.44 ± 0.02 ns (n = 9)  FLIM NKH/IBMX: 2.50 ± 0.02 ns (n = 9)  FLIM 5 μM cAMP: 2.47 ± 0.05 ns (n = 8) | |  |
| mlCNBD-FRET in sperm | cAMP: 103 ± 31 nM (n = 7) | | FLS basal: 2.0 ± 0.1 ns (n = 4)  FLS 6 μM cAMP: 2.3 ± 0.03 ns (n = 3) | |  |
|  | |  | |  | |

**Figure 5 - source data 1**
