# Supplementary material for: A novel biosensor to study cAMP dynamics in cilia and flagella
Source: eLife. 2016 Mar 22;5:e14052. doi: 10.7554/eLife.14052 (PMC4811770; doi:10.7554/eLife.14052)
Supplement: Figure 5—source data 2. — DOI: http://dx.doi.org/10.7554/eLife.14052.011 [file elife-14052-fig5-data2.docx]

| cAMP sensor | Design | cAMP  affinity | Reference |
| --- | --- | --- | --- |
| FlCRhR | Tetrameric PKA | 90 nM | (Adams et al, 1991) |
| R-CFP/C-YFP | Tetrameric PKA | 0.3 µM | (Mongillo et al, 2004) |
| R_R230K_-CFP/C-YFP | Mutant tetrameric PKA | 31.3 µM | (Mongillo et al, 2004) |
| Wild-type CNGA2 | CNG channel | 36 µM | (Rich et al, 2001) |
| ∆61-90C460W/E583M | Mutant CNG channel | 15 µM | (Rich et al, 2001) |
| H30 | Mutant Epac 1 | 12.5 µM | (Terrin et al, 2006) |
| mpH30 | Mutant Epac 1 | 20 µM | (Terrin et al, 2006) |
| nlsH30 | Mutant Epac 1 | 17.5 µM | (Terrin et al, 2006) |
| CFP-Epac1-YFP | Epac1 | 50 µM | (Ponsioen et al, 2004) |
| Epac 1-camps | cAMP-binding domain from Epac 1 | 2.4 µM | (Nikolaev et al, 2004) |
| Epac 2-camps | cAMP-binding domain B from Epac 2 | 0.9 µM | (Nikolaev et al, 2004) |
| PKA-camps | cAMP-binding domain B from PKA | 1.9 µM | (Nikolaev et al, 2004) |
| HCN2-camps | cAMP-binding domain from HCN 2 | 5.9 µM | (Nikolaev et al, 2006) |
| ICUE1/2 | CFP-Epac1/2-Citrine | 10-50 µM | (DiPilato et al, 2004; Violin et al, 2008) |
|  |  |  |  |

**Figure 5 - source data 2**
